# Myeloid neoplasms arising after methotrexate therapy for autoimmune rheumatological diseases do not exhibit poor-risk molecular features

**DOI:** 10.1038/s41408-024-01093-9

**Published:** 2024-07-19

**Authors:** Mihir D. Wechalekar, Lin-Pierre Zhao, Monika M. Kutyna, Lih En Hong, Joule Li, Kevin Hung, Hamish S. Scott, Anna Brown, Christopher C. Hahn, Karin Kassahn, Dariusz Ladon, David T. Yeung, Daniel Thomas, Mrinal Patnaik, Susanna Proudman, Lionel Ades, Mithun V. Shah, Chung H. Kok, Devendra K. Hiwase

**Affiliations:** 1 Rheumatology Unit, Flinders Medical Centre, Adelaide, Australia; 2 Rheumatology Unit, Royal Adelaide Hospital, Adelaide, Australia; 3 College of Medicine and Public Health, Flinders University, Adelaide, Australia; 4 Department of Hematology, Hôpital Saint-Louis, Université de Paris, Assistance Publique des Hôpitaux de Paris (AP-HP), Paris, France; 5 Université de Paris, Institut de Recherche Saint-Louis, EMiLy, INSERM U1160 Paris, France; 6 CNRS, GDR3697 “Microenvironment of tumor niches-Micronit”, Paris, France; 7 Haematology Department, Central Adelaide Local Health Network, Adelaide, Australia; 8 Blood Cancer, Precision Medicine Theme, South Australian Health and Medical Research Institute, Adelaide, Australia; 9 Adelaide Medical School, University of Adelaide, Adelaide, Australia; 10 Department of Medicine, Royal Adelaide Hospital, Adelaide, Australia; 11 Centre for Cancer Biology, SA Pathology and University of South Australia, Adelaide, Australia; 12 Genetics and Molecular Pathology, SA Pathology, Adelaide, Australia; 13 Division of Hematology, Mayo Clinic, Rochester, MN USA; 14 Université de Paris, Institut de Recherche Saint-Louis, INSERM U944 Paris, France

**Keywords:** Myelodysplastic syndrome, Translational research

**TO THE EDITOR:**

Autoimmune diseases, including autoimmune rheumatic diseases (AIRD) are reported in 8–30% of patients with myeloid neoplasm (MN), including myelodysplastic syndrome (MDS), chronic myelomonocytic leukemia (CMML), acute myeloid leukemia (AML) and myeloproliferative neoplasms (MPN) [1–4], and may precede or follow autoimmune disease. Despite increased recognition of the association, causative factors remain unresolved, and clinical outcomes are not established. For example, the role of cytotoxic and disease-modifying antirheumatic drugs (DMARDs) in the development of MN remains uncertain. DMARDs can be broadly classified as cytotoxic, immunomodulatory, or biologic. High-dose methotrexate, cyclophosphamide, and azathioprine are considered cytotoxic DMARDs [5], although the low-dose methotrexate used for AIRDs is generally considered to have an anti-inflammatory rather than cytotoxic mechanism of action [6]. Early reports suggested an increased incidence of MN following exposure to low-dose methotrexate, but this was not replicated in a recent case-control study [5]. Increased incidence of MN following treatment with azathioprine [5] and cyclophosphamide [6] was reported. However, these studies did not investigate the genetic profiling of MN following exposure to methotrexate and other DMARDs.

**Fig. 1:**
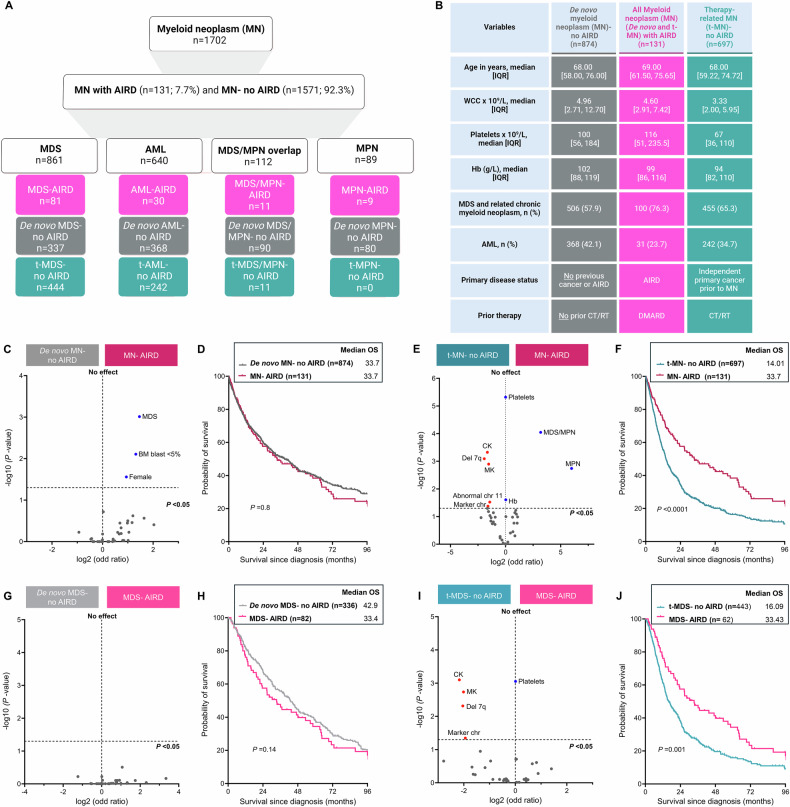
The cytogenetic and molecular profiles of myeloid neoplasms (MN) with autoimmune rheumatic diseases (AIRD) are comparable to de novo MN but distinct from therapy-related MN (t-MN) without AIRD. **A** Distribution of therapy-related myelodysplastic syndrome (t-MDS), therapy-related acute myeloid leukemia (t-AML), t-MDS/myeloproliferative neoplasm (MPN), de novo MDS, de novo AML, de novo MDS/MPN overlap, and MN with AIRD (MN-AIRD). **B** The clinical features that differentiate between the MN groups. Importantly, MN-AIRD included MN cases with AIRD irrespective of prior exposure to disease-modifying anti-rheumatic drugs (DMARD), t-MN no AIRD included MN patients with prior exposure to cytotoxic therapies for the treatment of independent primary cancer(s), and de novo MN no AIRD included cases of MN without AIRD. **C** Volcano plot comparing the clinical and mutation profile of MN-AIRD vs. de novo MN without AIRD. **D** Kaplan–Meier survival estimate comparing MN-AIRD vs. de novo MN without AIRD. **E** Volcano plot comparing clinical and mutation profile of MN-AIRD vs. t-MN without AIRD. **F** Kaplan–Meier survival estimate comparing MN-AIRD vs. t-MN without AIRD. **G** Volcano plot comparing the clinical and mutation profile of MDS-AIRD vs. de novo MDS without AIRD. **H** Kaplan–Meier survival estimate comparing MDS-AIRD vs. de novo MDS without AIRD. **I** Volcano plot comparing the clinical and mutation profile of MDS-AIRD vs. t-MDS without AIRD. **J** Kaplan–Meier survival estimate comparing MDS-AIRD vs. t-MDS without AIRD. *P*-values shown in all the volcano plots were corrected for multiple testing with the Benjamini–Hochberg (BH) procedure when appropriate. BM bone marrow, Del deletion, chr chromosome, Hb hemoglobin, CK complex karyotype, MK monosomal karyotype.

To address these gaps, we evaluated the burden of well-defined AIRD in a large international cohort of MN (*n* = 1702) managed in the South Australia Local Health Network (Adelaide, Australia; *n* = 1272) and the Mayo Clinic (Rochester, USA; *n* = 430). MDS (*n* = 861) was the most prevalent MN, followed by AML (*n* = 640), MDS/MPN (*n* = 112), and MPN (*n* = 89) (Fig. 1A and B). Cases of AIRD were identified by screening comorbidities reported in discharge summaries and clinic correspondence using International Classification of Diseases (ICD) codes (Supplementary Table 1).

The median age was 68 years (interquartile range, IQR 59–75), and 1023 (60%) patients were male. The median follow-up was 71.81 (IQR 30.6, 151.1) months and median overall survival (OS) was 23.8 months from the time of MN diagnosis (95% confidence interval, CI 22.2–26.1) with survival differences in MDS, AML and MPN (Supplementary Fig. 1A and B).

Overall, 7.7% of MN patients had AIRD (MN-AIRD). Although all MN subtypes were associated with AIRD, enrichment of AIRD was observed in patients with MDS, MDS/MPN, and MPN compared to AML (9.5% vs. 4.7%; *P* = 0.002). The most prevalent AIRD was rheumatoid arthritis (RA; *n* = 55, 42%) followed by autoimmune connective tissue diseases (CTD; *n* = 23, 17.6%), polymyalgia rheumatica (*n* = 18, 13.7%), vasculitis (*n* = 16, 12.2%), and peripheral and axial spondyloarthropathy (*n* = 9, 6.9%).

The AIRD prevalence rate of 7.7% in our cohort is lower than other reports of 8–30% [1–4]. This variation is likely attributed to differences in inclusion criteria. Our study analyzed well-defined AIRD cases, whereas other studies [1–4] have encompassed a broader range of autoimmune conditions including both rheumatological and non-rheumatological conditions, such as inflammatory bowel disease, autoimmune thyroiditis, and autoimmune thrombocytopenia. Furthermore, some studies included patients with asymptomatic immunological laboratory abnormalities (of unclear clinical relevance), as well as those exhibiting inflammatory (*vs*. autoimmune) manifestations or undifferentiated autoimmune diseases [3, 7]. It is also worth noting that many retrospective studies rely on registry-based data and case-mix analyses, utilizing ICD codes for classification. These methodologies introduce additional variability and potential biases in the reported findings.

The survival of MN with AIRD is also actively debated, with some studies reporting longer OS of MDS with autoimmune diseases (vs. MDS without autoimmune diseases) [1, 8], while others observe similar [3, 4, 9, 10], or poorer [2, 11, 12] OS. These conflicting results [1, 3] can be attributed to the inclusion of asymptomatic immunological serological abnormalities [7]. Secondly, although MN is highly heterogenous, the majority of the studies lack details on the subtype of MN.

We compared the clinical findings, cytogenetics, and survival of MN-AIRD vs. MN without AIRD. MN following exposure to cytotoxic therapies for other independent cancers, AIRD, or solid organ transplant are defined as therapy-related myeloid neoplasm (t-MN) (Fig. 1A and B); whereas MN without a preceding history of cytotoxic exposure is known as de novo MN. In our cohort, 54.2% (*n* = 923) patients were classified as de novo MN while 45.8% (*n* = 779) were classified as t-MN. Most patients with t-MN had undergone cytotoxic therapy for independent cancer (89.7%; *n* = 697). However, 10.3% (*n* = 82) of patients had therapies for AIRD or other autoimmune diseases.

The majority of clinical and molecular features were similar between de novo MN without AIRD and MN-AIRD, except for the enrichment of low blast MDS and females in MN-AIRD (Fig. 1C). The median OS of MN-AIRD was comparable to de novo MN without AIRD (33.7 vs. 33.7 months, *P* = 0.8; Fig. 1D). Chromosomal abnormalities portending poor prognosis, including complex karyotype (CK), monosomal karyotype (MK), deletion 7q (del 7q) and marker chromosome were enriched in t-MN following independent primary cancer (t-MN without AIRD) compared to MN-AIRD (Fig. 1E) and were associated with significantly poor survival in t-MN without AIRD compared to MN-AIRD (14.0 vs. 33.7 months; *P* < 0.0001; Fig. 1F**)**.

We then compared the clinical features and survival of cases with and without AIRD within the MN disease categories. The clinical features, cytogenetics, mutation profile, and survival of MDS-AIRD were similar to de novo MDS without AIRD (Fig. 1G, H). However, poor-risk cytogenetic changes were prevalent in t-MDS following treatment of primary cancer (Fig. 1I). In line with these distinct clinical features, we observed significantly poor survival in t-MDS without AIRD compared to MDS-AIRD (16.1 vs. 33.4 months, *P* = 0.001; Fig. 1J). We also observed a significant OS difference in MPN with and without AIRD (*P* = 0.02; Supplementary Fig. 1C), but no survival difference between MDS/MPN overlap with or without AIRD (*P* = 0.21; Supplementary Fig. 1D). Similarly, clinical features and median OS of de novo AML and t-AML without AIRD were similar to AML-AIRD (Supplementary Fig. 1E–H). Collectively, our study is one of the largest comprehensive analyses demonstrating that the clinical, cytogenetic, and molecular features and median OS of MN-AIRD are comparable to de novo MN without AIRD but highly distinct from t-MN without AIRD.

**Fig. 2:**
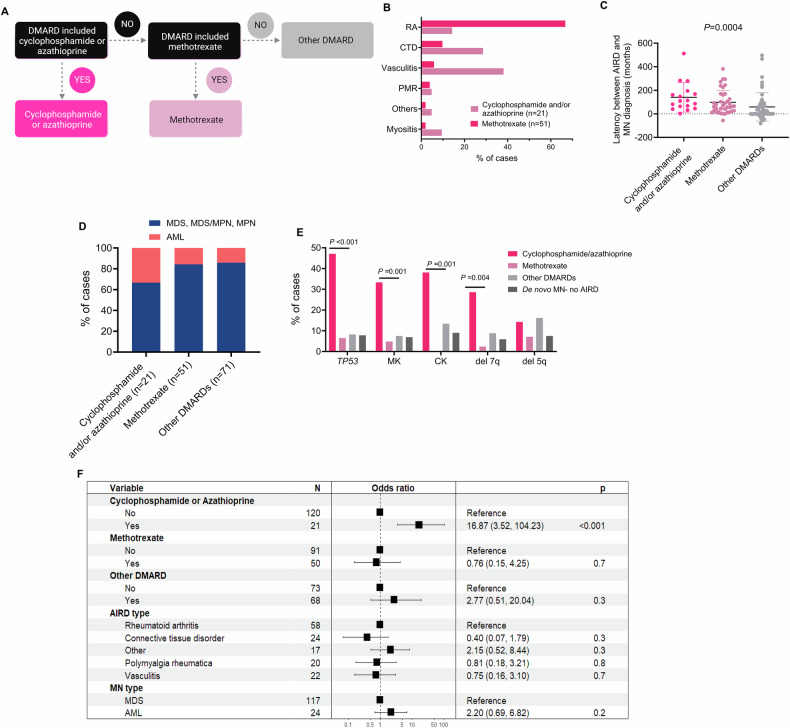
Myeloid neoplasm (MN) following exposure to low-dose methotrexate to treat AIRD is not associated with poor-risk cytogenetic and molecular profiles. **A** The hierarchical model is used to classify patients according to the disease-modifying antirheumatic drugs (DMARDs) used to treat autoimmune rheumatic diseases (AIRD). **B** The majority of cases treated with cyclophosphamide and/or azathioprine had vasculitis or autoimmune connective tissue diseases, while RA was the most prevalent AIRD in methotrexate-treated patients. **C** The interval between AIRD and MN diagnosis (latency) was significantly longer for patients treated with cyclophosphamide and/or azathioprine compared to methotrexate and other DMARDs. **D** The majority of patients treated with methotrexate and other DMARDs presented as myelodysplastic syndrome (MDS) whereas 30% of cyclophosphamide-treated patients presented as acute myeloid leukemia (AML). **E** There was striking enrichment of poor-risk cytogenetic changes in MN-AIRD treated with cyclophosphamide and/or azathioprine compared to methotrexate and biologic DMARDs. Cytogenetic data was available for the majority of cases treated with cyclophosphamide and/or azathioprine (*n* = 21), methotrexate (*n* = 42), other DMARD (*n* = 68), and de novo MDS without AIRD (*n* = 474). Similarly, *TP53* mutations were available for the majority of cases treated with cyclophosphamide and/or azathioprine (*n* = 17), methotrexate (*n* = 31), other DMARD (*n* = 49), and de novo MDS No AIRD (*n* = 370). **F** Multivariable logistic regression analysis demonstrating that cyclophosphamide/azathioprine therapy was associated with poor-risk features of MN after adjusting for MN subtype, and AIRD subtype. Del deletion, chr chromosome, Hb hemoglobin. Chi-squared test was used to determine associations between categorical variables.

Given that a proportion of patients received a combination of cytotoxic, immunomodulatory, and biologic DMARDs, we used a hierarchical model to classify patients according to the treatment received. For example, if patients received cyclophosphamide or azathioprine, either alone or in combination with other DMARDs, they were classified as having had prior exposure to cyclophosphamide and/or azathioprine, while patients who received methotrexate but not cyclophosphamide or azathioprine were classified as receiving methotrexate irrespective of other DMARDs, and patients who did not receive these three agents were grouped together under “other DMARDs” (Fig. 2A). We next evaluated if the type of DMARD influences the chromosomal, mutation and clinical presentations of MN.

Despite screening a large cohort of 1702 patients with MN, the number of MN-AIRD cases was relatively small (*n* = 131), hence, we included additional cases of MDS and CMML with AIRD (*n* = 51) managed at the Saint-Louis Hospital (Paris, France). Of the 182 MN-AIRD cases, AIRD treatment details were available for 152 (83.5%), of which 94.1% (*n* = 143) were treated with DMARDs. In this cohort, 14.7% (*n* = 21), 35.6% (*n* = 51), and 49.7% (*n* = 71) patients were treated with cyclophosphamide/azathioprine, methotrexate and other DMARDs (prednisolone, hydroxychloroquine, etc.), respectively (Supplementary Table 2). The majority (66.7%) of cases treated with cyclophosphamide/azathioprine were vasculitis (38.1%) and autoimmune CTD (28.6%), whereas RA was most prevalent AIRD in methotrexate-treated patients (*n* = 34; 66.7%; Fig. 2B). The latency between AIRD and MN diagnosis was significantly longer in cases managed with cyclophosphamide/azathioprine compared to methotrexate and other DMARDs (109.3 vs. 63.5 vs. 13.0 months; *P* = 0.0004; Fig. 2C).

The majority (92.5%) of MN-AIRD treated with methotrexate and other DMARDs presented as MDS and related MN. Conversely, 30% of MN-AIRD exposed to cyclophosphamide or azathioprine presented as AML (Fig. 2D). We also observed a significant difference in the cytogenetic and molecular profiles of MN following exposure to these agents. For instance, striking enrichment of CK, MK, del 7q/monosomy 7, del 5q/monosomy 5, marker chromosome, and *TP53* mutations were observed in MN-AIRD cases who received cyclophosphamide/azathioprine (Fig. 2E). This was further substantiated in a multivariable logistic regression analysis. Cyclophosphamide/azathioprine therapy was associated with poor-risk features of MN after adjusting for the MN subtype, and AIRD subtype (Fig. 2F). In contrast, these poor-risk disease features were observed in 2–4% of patients treated with methotrexate and were comparable to MN-AIRD managed with non-cytotoxic DMARDs and de novo MN (Fig. 2E, F). Furthermore, prior exposure to low-dose methotrexate has been excluded as a qualifying criterion for MN post-cytotoxic therapy in the revised 5th edition of the World Health Organization classification of MN [13] but not from the International Consensus Classification [14] of MN. AIRD subtype was not associated with poor risk features of MN or survival of MN-AIRD (Fig. 2F and Supplementary Fig. 2).

Due to the retrospective nature of the study, obtaining a detailed DMARD treatment duration was challenging. Despite the limitations, this is the largest study demonstrating that clinical features and survival of the majority of cases of MN-AIRD, especially following exposure to low-dose methotrexate, typically used to manage rheumatological diseases, are comparable to de novo MN and should not be considered a therapy-related neoplasm. As methotrexate is widely used as an anchor drug in many rheumatology treatment regimens, this is a significant and clinically relevant finding.

### Supplementary information

Supplementary Materials
