# Supplementary material for: Myeloid neoplasms arising after methotrexate therapy for autoimmune rheumatological diseases do not exhibit poor-risk molecular features
Source: Blood Cancer J. 2024 Jul 19;14(1):116. doi: 10.1038/s41408-024-01093-9 (PMC11271612; doi:10.1038/s41408-024-01093-9)
Supplement: Supplementary file 1 — Supplementary Materials [file 41408_2024_1093_MOESM1_ESM.docx]

**Supporting Information for:**

**Myeloid neoplasms arising after methotrexate therapy for autoimmune rheumatological diseases do not exhibit poor-risk molecular features**

**This section includes:**

1. **Supplemental Methods including references**
2. **Supplemental Tables 1 – 2**
3. **Supplemental Figures 1 - 2**

**Supplemental Methods**

**Clinical data**

The South Australian MDS/AML (SA-MDS/AML) registry stands as a comprehensive repository of MDS, AML, and MDS/MPN overlap cases, with curated clinical data and extensive long-term follow-up records. This collaborative initiative spans internationally, encompassing cases managed in South Australia (n=1272) and at the Mayo Clinic in Rochester, USA (n=430). Notably, all cases contributed by the Mayo Clinic are therapy-related myeloid neoplasms (t-MN) (n=430), while the majority of cases from the South Australian MDS/AML registry are *de novo* myeloid neoplasms (n=922; 73%), with t-MN cases occurring at the anticipated frequency (n=350; 23%). A dedicated team from both centers has meticulously curated both electronic and paper records of all patients diagnosed with myeloid neoplasms (MN) according to the World Health Organization 2016 classification. To ensure accuracy, data regarding autoimmune rheumatic diseases (AIRD) diagnoses underwent screening using case-mix data employing International Classification of Disease codes (*Supplemental Table 1*). Rheumatologists (LH and MW) meticulously reviewed the rheumatology database, clinic letters, paper records, electronic medical records, and laboratory investigations to validate the diagnosis and management of AIRD within the South Australian cohort.

DMARDs can be broadly classified as cytotoxic, immunomodulatory, or biologic. Cytotoxic DMARDS include high-dose methotrexate, cyclophosphamide, azathioprine, mercaptopurine, and mitoxantrone. Immunomodulatory DMARDs include low-dose methotrexate, mycophenolate mofetil, leflunomide, sulfasalazine, hydroxychloroquine, and cyclosporine. Biologic DMARDs are highly specific and target a specific pathway of the immune system^1^, and are classified into two main groups: (i) monoclonal, chimeric, and humanized antibodies, for example, TNF inhibitors (etanercept, infliximab, adalimumab and golimumab), IL-1 inhibitor (anakinra), human IgG1λ recombinant monoclonal antibody directed against B lymphocyte stimulator (belimumab), anti-CD20 antibody (rituximab), and IL-6 blocker (tocilizumab); or (ii) small molecules targeting Janus kinases (abatacept, tofacitinib and ruxolitinib).

All patients were allocated individual identifiers and anonymized. Data collection encompassed:

(i) Comprehensive information regarding the AIRD, including treatments, age at diagnosis, and the interval between AIRD and MN diagnosis (referred to as the latency period). (ii) Analysis of blood counts, bone marrow blasts, conventional G-banding karyotypes, pathogenic somatic mutations, and the WHO subtype of MN. (iii) Assessment of clinical outcomes, encompassing time of death from any cause, patient loss to follow-up, and the occurrence of AML transformation.

**Next-generation sequencing panels**

DNA was extracted from bone marrow aspirates and sequencing was performed using a targeted next-generation sequencing (NGS) panel at each institution as described previously^2,3^. The following frequently mutated genes in myeloid malignancies were analyzed: *ASXL1, BCOR, CBL, CEBPA, DDX41, DNMT3A, EZH2, FLT3, GATA2, IDH1, IDH2, JAK2, KIT, KRAS, MPL, NPM1, NRAS, PTPN11, RUNX1, SETBP1, SF3B1, SRP72, SRSF2, STAG2, TERT, TET2, TP53, U2AF1, WT1,* and *ZRSR2.* Pathogenic variants with variant allele frequency (VAF) ≥2%, were retained for further analysis. Classification of variants as pathogenic or likely pathogenic was performed as previously described.

**Statistical analysis**

Comparisons between groups for numerical variables were performed using Mann-Whitney U-test or Student’s t test. Chi-squared or Fisher’s exact test was used to determine associations between categorical variables. Univariate logistic regression was used to compare between groups and determine the odds ratio and *P* values were adjusted for the multiple testing using Benjamini-Hochberg correction. Multivariate logistic regression analysis was used to evaluate factors associated with AIRD. *P* values <0.05 were considered statistically significant. All statistical analyses were conducted using R v.4.1.1 and GraphPad Prism v9.

**Supplementary References**

1. Ertz-Archambault N, Kosiorek H, Taylor GE, et al. Association of Therapy for Autoimmune Disease With Myelodysplastic Syndromes and Acute Myeloid Leukemia. *JAMA oncology*. Jul 1 2017;3(7):936-943. doi:10.1001/jamaoncol.2016.6435

2. Singhal D, Wee LYA, Kutyna MM, et al. The mutational burden of therapy-related myeloid neoplasms is similar to primary myelodysplastic syndrome but has a distinctive distribution. *Leukemia*. Dec 2019;33(12):2842-2853. doi:10.1038/s41375-019-0479-8

3. Hiwase D, Hahn C, Tran ENH, et al. TP53 mutation in therapy-related myeloid neoplasm defines a distinct molecular subtype. *Blood*. Mar 2 2023;141(9):1087-1091. doi:10.1182/blood.2022018236

**Supplementary Tables**

**Supplemental Table 1. International Classification of Diseases 10^th^ Revision (ICD 10) codes**

| Major category | ICD 10 code |
| --- | --- |
| Rheumatoid arthritis | **M05 Seropositive rheumatoid arthritis:**   - M05.0 Felty syndrome - M05.1 Seropositive rheumatoid arthritis: Rheumatoid lung disease - M05.2 Rheumatoid vasculitis - M05.3 Rheumatoid arthritis with involvement of other organs and systems - M05.8 Other seropositive rheumatoid arthritis - M05.9 Seropositive rheumatoid arthritis, unspecified   **M06 Other rheumatoid arthritis:**   - M06.0 Seronegative rheumatoid arthritis - M06.1 Adult-onset Still disease - M06.2 Rheumatoid bursitis - M06.3 Rheumatoid nodule - M06.4 Inflammatory polyarthropathy - M06.8 Other specified rheumatoid arthritis - M06.9 Rheumatoid arthritis, unspecified |
| Peripheral and axial spondyloarthritis | **M07 Psoriatic and enteropathic arthropathies:**   - M07.0 Distal interphalangeal psoriatic arthropathy - M07.1 Arthritis mutilans - M07.2 Psoriatic spondylitis - M07.3 Other psoriatic arthropathies - M07.4 Arthropathy in Crohn disease [regional enteritis] - M07.5 Arthropathy in ulcerative colitis - M07.6 Other enteropathic arthropathies - L40.5 Arthropathic psoriasis   **M45 Ankylosing spondylitis**  **M46.0 Spinal enthesopathy:**   - M46.1 Sacroiliitis, not elsewhere classified - M46.8 Other specified inflammatory spondylopathies - M46.9 Inflammatory spondylopathy, unspecified   **M48.9 Spondylopathy, unspecified** |
| Juvenile idiopathic arthritis | **M08 Juvenile arthritis:**   - M08.0 Juvenile rheumatoid arthritis - M08.1 Juvenile ankylosing spondylitis - M08.2 Juvenile arthritis with systemic onset - M08.3 Juvenile polyarthritis (seronegative) - M08.4 Pauciarticular juvenile arthritis - M08.8 Other juvenile arthritis - M08.9 Juvenile arthritis, unspecified   **M09 Juvenile arthritis in diseases classified elsewhere:**   - M09.0 Juvenile arthritis in psoriasis - M09.1 Juvenile arthritis in Crohn disease [regional enteritis] - M09.2 Juvenile arthritis in ulcerative colitis - M09.8 Juvenile arthritis in other diseases classified elsewhere |
| Undifferentiated inflammatory arthritis | - M36.1 Arthropathy in neoplastic disease - M36.4 Arthropathy in hypersensitivity reactions classified elsewhere - M79.0 Rheumatism, unspecified - M12.0 Chronic postrheumatic arthropathy [Jaccoud] |
| Small vessel vasculitis | **M30 Polyarteritis nodosa and related conditions:**   - M30.0 Polyarteritis nodosa - M30.1 Polyarteritis with lung involvement [Churg-Strauss] - M30.2 Juvenile polyarteritis - M30.8 Other conditions related to polyarteritis nodosa   **M31 Other necrotizing vasculopathies:**   - M31.0 Hypersensitivity angiitis - M31.1 Thrombotic microangiopathy - M31.2 Lethal midline granuloma - M31.3 Wegener granulomatosis - M31.7 Microscopic polyangiitis - M31.8 Other specified necrotizing vasculopathies - M31.9 Necrotizing vasculopathy, unspecified |
| Large vessel vasculitis | - M31.4 Aortic arch syndrome [Takayasu] - M31.5 Giant cell arteritis with polymyalgia rheumatica - M31.6 Other giant cell arteritis |
| Inflammatory rheumatic disorders | - M35.3 Polymyalgia rheumatica |
| Systemic lupus erythematosus | **M32 Systemic lupus erythematosus:**   - M32.0 Drug-induced systemic lupus erythematosus - M32.1 Systemic lupus erythematosus with organ or system involvement - M32.8 Other forms of systemic lupus erythematosus - M32.9 Systemic lupus erythematosus, unspecified |
| Systemic sclerosis | **M34 Systemic sclerosis:**   - M34.0 Progressive systemic sclerosis - M34.1 CR(E)ST syndrome - M34.2 Systemic sclerosis induced by drugs and chemicals - M34.8 Other forms of systemic sclerosis - M34.9 Systemic sclerosis, unspecified - M35.1 Other overlap syndromes   **M36.8 Systemic disorders of connective tissue in other diseases** classified elsewhere |
| Sjogren’s syndrome | - M35.0 Sicca syndrome [Sjögren] |
| Inflammatory myositis | **M33 Dermatopolymyositis:**   - M33.0 Juvenile dermatomyositis - M33.1 Other dermatomyositis - M33.2 Polymyositis - M33.9 Dermatopolymyositis, unspecified   **M36.0 Dermato(poly)myositis in neoplastic disease**  **M60.1 Interstitial myositis**  **M63.3 Myositis in sarcoidosis**  **M35.4 Diffuse (eosinophilic) fasciitis**  **G72.4 Inflammatory myopathy, not elsewhere classified** |
| Auto-inflammatory disorders | - M35.2 Behçet disease |
| Granulomatous inflammation | - D86.8 Sarcoidosis of other and combined sites - D86.9 Sarcoidosis, unspecified |

**Supplementary Table 2. DMARD group comparison**

| Variables | Cyclophosphamide/ Azathioprine  *n* = 21 | Methotrexate  *n* = 51 | Other DMARD  *n* = 71 | *P*- value |
| --- | --- | --- | --- | --- |
| Clinical features at MN diagnosis |  | | | |
| Age, median [IQR] | 64.00 [61.00, 74.50] | 68.90 [60.10, 75.30] | 72.00 [64.95, 79.00] | 0.092 |
| Gender n (%):  Female  Male | 6 (28.6)  15 (71.4) | 34 (66.7)  17 (33.3) | 42 (59.2)  29 (40.8) | 0.011 |
| WCC x 10^9^/L, median [IQR] | 4.79 [3.60, 8.10] | 5.16 [3.35, 8.25] | 4.60 [2.50, 7.79] | 0.496 |
| Platelets x 10^9^/L, median [IQR] | 111 [53, 199] | 118 [62, 237] | 135.00 [60, 218] | 0.936 |
| Hb (g/L), median [IQR] | 98 [81, 122] | 101 [91, 115] | 96 [86.5, 121] | 0.88 |
| Monocytes x 10^9^/L, median [IQR] | 0.50 [0.20, 0.81] | 0.43 [0.23, 0.79] | 0.63 [0.33, 1.23] | 0.533 |
| Neutrophils x 10^9^/L, median [IQR] | 4.00 [3.10, 5.20] | 4.20 [1.80, 5.55] | 3.90 [2.85, 5.70] | 0.918 |
| Myeloid Neoplasm disease subtype, n (%):  MDS  AML  MDS/MPN overlap  MPN | 13 (61.9)  7 (33.3)  1 (4.8)  0 (0.0) | 34 (66.7)  8 (15.7)  6 (11.8)  3 (5.9) | 46 (64.8)  10 (14.1)  12 (16.9)  3 (4.2) | 0.34 |
| BM blasts, n (%): <5% 5-9% 10-19% ≥20% | 10 (47.6)  3 (14.3)  1 (4.8)  7 (33.3) | 29 (63.0)  6 (13.0)  4 (8.7)  7 (15.2) | 41 (57.7)  13 (18.3)  7 (9.9)  10 (14.1) | 0.513 |

**Supplementary Figure S1**

**
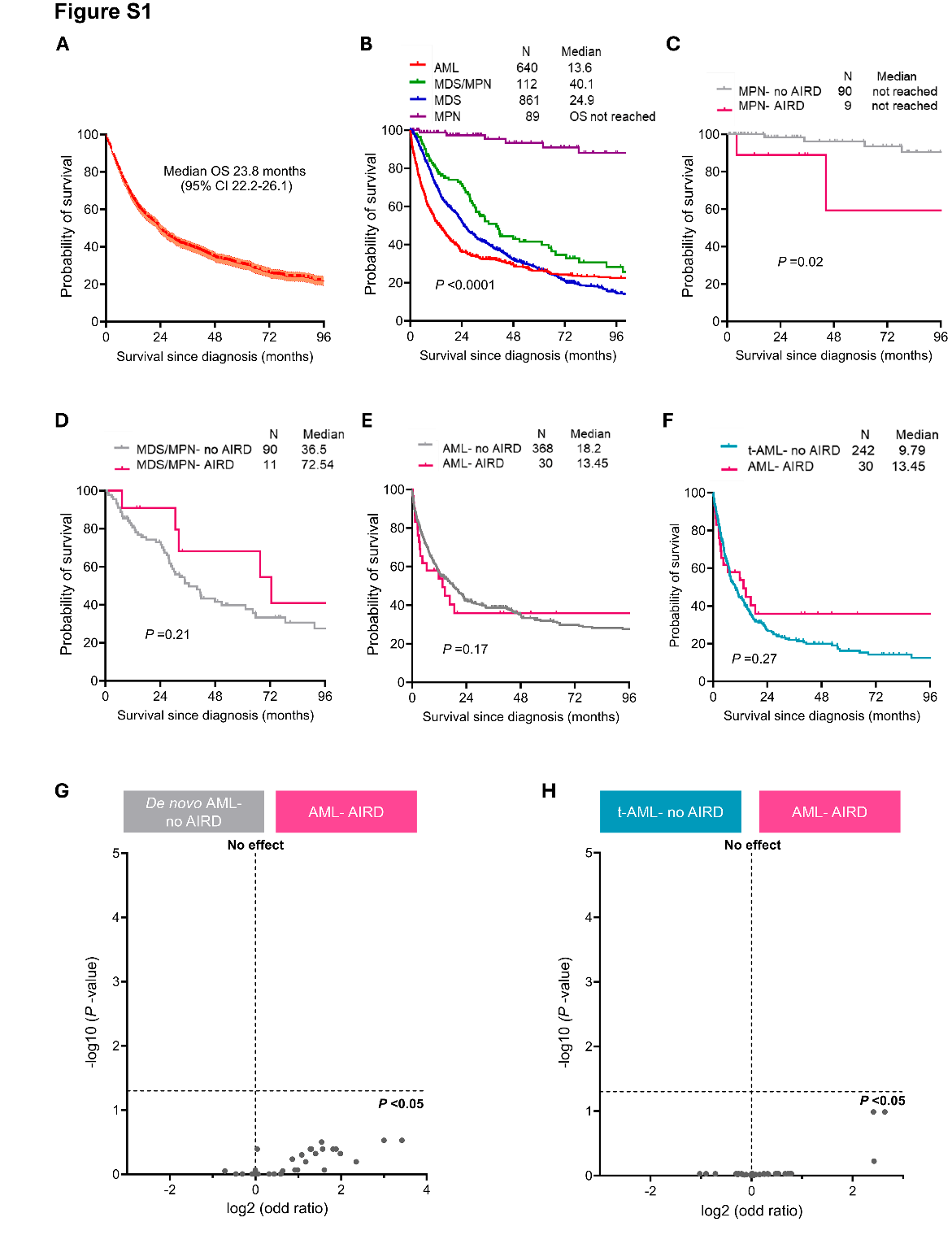
**

**Figure S1.** Overall survival (OS) of the (A) whole myeloid neoplasm cohort, and according to (B) MN subtype; (C) MPN with and without AIRD; (D) MDS/MPN overlap with and without AIRD; (E) *de novo* AML with and without AIRD; and (F) t-AML with and without AIRD; (G) Volcano plot comparing clinical and mutation profile of AML-AIRD *vs.* *de novo* AML without AIRD; (H) Volcano plot comparing clinical and mutation profile of AML-AIRD *vs.* t-AML without AIRD. *P*-values shown in all the Volcano plot was corrected for multiple testing with the Benjamini-Hochberg (BH) procedure when appropriate.

**Supplementary Figure S2**

**
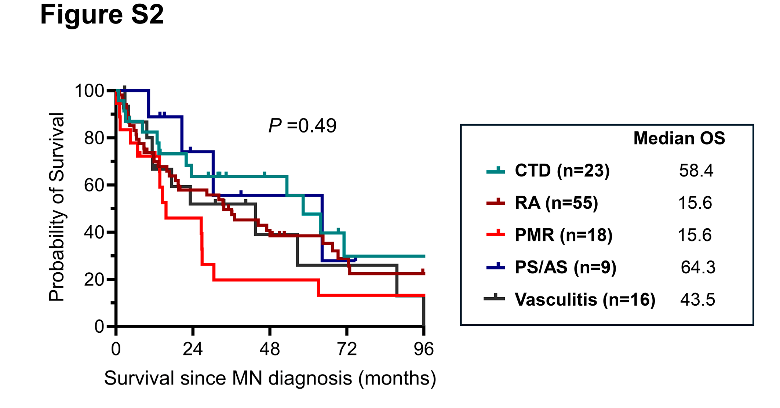
**

**Figure S2. Overall survival (OS) of the myeloid neoplasm with AIRD (MN-AIRD) according to AIRD subtypes.** *Abbreviations: CTD,* inflammatory connective tissue diseases*; RA, rheumatoid arthritis; PMR,* polymyalgia rheumatica*; PS/AS,* peripheral and axial spondyloarthropathy*.*
